# Identity-by-descent estimation with population- and pedigree-based imputation in admixed family data

**DOI:** 10.1186/s12919-016-0046-5

**Published:** 2016-10-18

**Authors:** Mohamad Saad, Alejandro Q. Nato, Fiona L. Grimson, Steven M. Lewis, Lisa A. Brown, Elizabeth M. Blue, Timothy A. Thornton, Elizabeth A. Thompson, Ellen M. Wijsman

**Affiliations:** 1 Department of Biostatistics, University of Washington, Seattle, WA USA; 2 Division of Medical Genetics, Department of Medicine, University of Washington, Seattle, WA USA; 3 Department of Statistics, University of Washington, Seattle, WA USA; 4 Department of Genome Sciences, University of Washington, Seattle, WA USA

## Abstract

**Background:**

In the past few years, imputation approaches have been mainly used in population-based designs of genome-wide association studies, although both family- and population-based imputation methods have been proposed. With the recent surge of family-based designs, family-based imputation has become more important. Imputation methods for both designs are based on identity-by-descent (IBD) information. Apart from imputation, the use of IBD information is also common for several types of genetic analysis, including pedigree-based linkage analysis.

**Methods:**

We compared the performance of several family- and population-based imputation methods in large pedigrees provided by Genetic Analysis Workshop 19 (GAW19). We also evaluated the performance of a new IBD mapping approach that we propose, which combines IBD information from known pedigrees with information from unrelated individuals.

**Results:**

Different combinations of the imputation methods have varied imputation accuracies. Moreover, we showed gains from the use of both known pedigrees and unrelated individuals with our IBD mapping approach over the use of known pedigrees only.

**Conclusions:**

Our results represent accuracies of different combinations of imputation methods that may be useful for data sets similar to the GAW19 pedigree data. Our IBD mapping approach, which uses both known pedigree and unrelated individuals, performed better than classical linkage analysis.

## Background

In the last few years, imputation approaches have been widely used in genetic studies, especially in the context of genome-wide association studies and population-based designs. This is a result of an attractive feature of imputation: it increases genetic information at low-cost. Moreover, imputation allows meta-analysis between different studies, especially when different genotyping platforms are used. The main idea of imputation is to: (a) genotype or sequence a small subset of individuals on a dense single nucleotide polymorphism (SNP) panel, (b) genotype remaining individuals on a sparse SNP panel, and (c) use statistical methods for imputation, generally based on hidden Markov models, to impute untyped SNPs from the dense panel into these individuals with only sparse (or no) genotyped SNPs.

Imputation approaches have been proposed for both family- and population-based designs. However, the performance of these approaches has not yet been thoroughly evaluated and compared in pedigree data. The availability of the International Haplotype Map Project (HapMap) [1] and the 1000 Genomes Project [2] have allowed population-based imputation to become widespread. However, these databases are not ideal for family-based imputation methods, which require that the dense SNP panel individuals be selected from pedigrees. In addition, imputation approaches for large pedigrees are more challenging, and only quite recently has an approach been proposed and implemented in the program called Genotype Imputation Given Inheritance (GIGI) [3] that is able to efficiently handle large pedigrees and accurately impute rare variants. The search for rare risk variants has made imputation in family-based designs more important, because of enrichment of such variants in relatively large pedigrees [4] since linkage analysis may be powerful for initiating rare variant identification. An attractive study design is to (a) perform linkage analysis, (b) perform imputation in the region(s) with linkage signals, and (c) perform association analysis on the imputation data in these regions. It is well-established that larger pedigrees are advantageous for this linkage analysis component.

Identity-by-descent (IBD) underpins both linkage analysis and imputation. For linkage analysis, once IBD has been determined, the pedigree structure is no longer needed for subsequent computations. For imputation, IBD within pedigrees or (ancestrally) across individuals is used as a source of correlation between individuals for family- and population-based imputation.

In this study, we evaluated and compared the performance of several family- and population-based imputation approaches in the Mexican American pedigree data provided by Genetic Analysis Workshop 19 (GAW19). We also evaluated the effect on imputation accuracy of the number of selected individuals for sequencing and the way they were selected, randomly or by tailored selection with GIGI-Pick [5]. In addition, we evaluated the performance of a new IBD mapping approach that we propose, which combines IBD information inferred (a) using a sparse SNP panel from known pedigrees, and (b) using a dense SNP panel from unrelated individuals across pedigrees.

## Methods

### Data

All our analyses used chromosome 3. For population-based imputation, we used the whole genome sequence data on the 464 sequenced individuals in a region of 2.5 Mbp (Marker Set 1 [MS-1]; Table 1). These individuals belong to 16 pedigrees. For the purpose of IBD estimation within pedigrees, required for pedigree-based imputation, we used a set of 351 well-spaced SNPs (Marker Set 2 [MS-2]; Table 1) extracted from the SNP data of all genotyped individuals in these 16 pedigrees. Physical positions were converted to meiotic map positions (cM) by reference to the Rutgers map [6], and converted to positions based on the Haldane map function. For IBD mapping analysis based on a meiotic map, with the knowledge of the trait simulation model provided in the “Answers” file obtained with the data, MS-2 was used to compute IBD within pedigrees. We focused the IBD mapping analysis on 7 pedigrees (Pedigrees 05, 06, 07, 08, 10, 21, 25; total number of 529 individuals) that showed high levels of relatedness on the basis of Genetic Analysis Workshop 18 analysis [7]. We selected 17 pairs of individuals (21 distinct individuals) who had shown high levels of pair-wise kinship and were not related by the pedigree information. For these 21 cryptically related individuals, we used Marker Set 3 (MS-3) (Table 1) to infer IBD among “unrelated” individuals.

**Table 1:** Marker sets

Marker sets	Number of SNPs	Relevant information
MS-1	~15,000	chr 3: 46,750 Kbp–49,250 Kbp
MS-2	351	Mean spacing ~0.64 cM; in linkage equilibrium
MS-3	48,892	MAF > 0.05; genotype completion > 99 %

*chr* chromosome, *MAF* minor allele frequency

### Selection of dense SNP panel individuals for imputation

Among the 464 sequenced individuals, we chose to include 100 or 200 of them as if genotyped on the dense SNP panel. In proportion to the number of sequenced individuals in each pedigree, we selected individuals following 2 strategies: (a) random selection or (b) via GIGI-Pick (a method to help with informative choices) [5]. For GIGI-Pick, we used the genome-wide coverage option, which selects subjects based on the pedigree structure. In the group of the remaining 264 or 364 individuals, we extracted genotypes of SNPs present in the SNP chip of our region of interest (~700 SNPs), to form the set of sparse SNP panel individual data set.

### Population-based imputation

We performed 2-step imputation, with pre-phasing of markers in the first step, and imputation in the second step. The pre-phasing algorithms/programs we used were: BEAGLE [8], IMPUTE2 [9], MaCH [10], and SHAPEIT [11]. For SHAPEIT, we used 2 types of phasing. The first accounts for some pedigree structure information (called here SHAPEITped) while the second ignores the pedigree structure (SHAPEIT). Using the obtained phased genotypes, we performed imputation using the following algorithms/programs: BEAGLE, IMPUTE2, MaCH, minimac, and MaCHAdmix [12]. We implemented the following combinations of 2-step imputation (Pre-phasing–Imputation):

- BEAGLE–BEAGLE, SHAPEIT–BEAGLE, and SHAPEITped–BEAGLE;
- MaCH–MaCH, MaCH–minimac, SHAPEIT–minimac, and SHAPEITped–minimac;
- MaCH–MaCHAdmix, SHAPEIT–MaCHAdmix, and SHAPEITped–MaCHAdmix;
- IMPUTE2–IMPUTE2, SHAPEIT–IMPUTE2, and SHAPEITped–IMPUTE2.

### Family-based imputation

We performed pedigree-based imputation using GIGI [3]. This method uses inheritance vector (IV) realizations, reflecting the IBD flow in pedigrees, estimated on the sparse SNP panel (MS-2) for all individuals. The MORGAN/gl_auto program [13] was used to obtain these IV realizations. Based on these IV realizations, the dense SNP panel, the meiotic map, the allele frequencies of the dense SNPs, and the pedigree structure, GIGI infers the missing genotypes at untyped SNPs. Note that the first step (IBD estimation) is equivalent to the prephasing step used by population-based approaches.

### Combining family- and population-based imputation

We also combined imputation obtained by GIGI and population-based approaches via a flexible framework described elsewhere [14]. We compared 3 versions: (a) GIGI + SHAPEITped–BEAGLE, (b) GIGI + SHAPEITped–IMPUTE2, and (c) GIGI + SHAPEITped–minimac. As a metric of imputation accuracy, we calculated 2 mean correlation measures for all of the versions described above: (a) *ρ*_*1*_ = sum of correlation/total number of imputed SNPs (given minor allele frequency, MAF, in imputed data > 0), and (b) *ρ*_*2*_ = sum of correlation/total number of imputed SNPs in the reference panel (given MAF in reference panel data > 0). Correlation was computed for each SNP between the true and the imputed genotypes in the sparse SNP panel individuals, and then averaged across all imputed SNPs. As a metric of imputation accuracy, the use of correlation, which implicitly adjusts for the MAF of SNPs, is better than the use of concordance, which gives misleading results for rare variants. Other existing measures could also be used but there is no clear consensus in the literature of which performs best in all situations. In any case, our aim was to compare the accuracy between approaches and not to evaluate the accuracy *per se* for each approach. In this case, the correlation measure provides the necessary information for this comparison for both rare and common variants.

### IBD-mapping analysis

We used a set of 1000 IBD graphs [15, 16] that had been realized on the 7 pedigrees by the MORGAN/gl_auto program using the MS-2 subset of SNPs. Using the MORGAN/ibd_haplo program and MS-3, we found that many of the 17 pairs of individuals share IBD in the range 50 to 75 cM on chromosome 3, and that all pairs gave a strong signal of IBD at 69 cM. To infer IBD between the cryptically related individuals, we used a new program, ibd_stitch [17], which permits jointly-consistent location-specific IBD to be inferred among multiple individuals. Using ibd_stitch, 1000 IBD graphs in compact format [17] were realized jointly on the 21 distinct individuals. We wrote new R code to merge the joint 21-individual IBD graphs on the cryptically related individuals, with the IBD graphs on the 7 pedigrees. The merging was done in 2 groups (pedigrees 05, 06, 21, 25 and 10, 08, 07), which were chosen so that the merged graph had 2 components with an approximately equal number of individuals, with no cryptically related pairs linking the two groups. The merging was performed at the 351 MS-2 SNP positions to give 1000 IBD graphs on the combined set of 529 individuals. These merged graphs thus included location-specific between-family IBD in addition to the pedigree IBD. Given IBD graphs realized conditional on SNP data, logarithm of the odds (LOD) scores can be computed without further reference to the pedigree structures or SNP data [15]. Additionally, the MORGAN/gl_lods program uses equivalence of IBD graphs across realizations and across locations [16], to ensure that each distinct LOD score contribution is computed once only.

We used the 200 simulated traits of diastolic blood pressure (DBP) with a causal gene *(MAP4)* at 69 cM (47,892,180 to 48,130,769 bp) on chromosome 3. Trait data were preadjusted for age, sex, and current use of antihypertensive medications, and we used a trait model previously developed [7] for this gene (quantitative trait locus model with parameters defined by the SNP with the biggest contribution to the simulated trait variance). Using the MORGAN/gl_lods program, we computed LOD scores at the MS-2 SNP positions, for all 200 traits, on each of the 7 component families and on the merged IBD graphs that included the between-family IBD. We can thus compare the LOD scores calculated from the IBD graphs containing pedigree data only, with the IBD graphs of merged pedigree and population IBD.

## Results

### Imputation accuracy

Our results for several MAF bins were: rare variants ∈ (0,0.01], uncommon variants ∈(0.01,0.15], and common variants ∈ (0.15,0.5]. Table 2 shows that the prephasing algorithm has great influence on the imputation accuracy for every MAF bin. The use of the SHAPEITped algorithm gave the best imputation performance for every imputation algorithm used, and for every MAF bin. For example, in the case of rare variants, when we used BEAGLE for imputation, the mean correlation (*ρ*_1_) increased by 0.07 when SHAPEITped was used for pre-phasing rather than BEAGLE. The increase is even more striking (~0.18) for the imputation using minimac and the pre-phasing using SHAPEITped rather than MaCH. Accounting for the pedigree structure information during pre-phasing (SHAPEITped) showed a slight but consistently better imputation accuracy for all approaches and MAF bins (increase of ~0.02; results not shown).

**Table 2:** Random selection of 200 reference/dense SNP panel individuals

	(0–0.01]: #SNPs = 4604 SNPs			(0.01–0.15]: #SNPs = 1765 SNPs			(0.15–0.5]: #SNPs = 979 SNPs		
Imputation approaches	#SNPe	ρ_1_	ρ_2_	#SNPe	ρ_1_	ρ_2_	#SNPe	ρ_1_	ρ_2_
BEAGLE-BEAGLE	4325	0.209	0.196	1673	0.587	0.556	976	0.897	0.895
SHAPEITped-BEAGLE	4554	0.270	0.267	1763	0.730	0.729	979	0.978	0.978
MaCH-MaCH	4388	0.332	0.316	1755	0.595	0.591	979	0.801	0.801
MaCH-minimac	4523	0.460	0.452	1763	0.706	0.706	979	0.910	0.910
SHAPEITped-minimac	4507	0.642	0.629	1765	0.894	0.894	979	0.985	0.985
MaCH-MaCHAdmix	4340	0.420	0.396	1754	0.687	0.683	979	0.896	0.896
SHAPEITped-MaCHAdmix	4352	0.527	0.498	1760	0.773	0.770	979	0.904	0.904
GIGI	3763	0.610	0.498	1719	0.557	0.542	979	0.581	0.581
IMPUTE2-IMPUTE2	4230	0.370	0.340	1735	0.660	0.649	978	0.898	0.897
SHAPEITped-IMPUTE2	4401	0.485	0.464	1741	0.713	0.703	979	0.923	0.923
GIGI + SHAPEITped-BEAGLE	4507	0.643	0.630	1764	0.790	0.789	979	0.972	0.972
GIGI + SHAPEITped-minimac	4507	0.693	0.678	1764	0.860	0.860	979	0.980	0.980
GIGI + SHAPEITped-IMPUTE2	4491	0.635	0.619	1746	0.740	0.732	979	0.876	0.876

#SNPs is the total number of SNPs in the reference panel; #SNPe is the number of imputed SNPs as polymorphic; ρ_1_ is the mean correlation of all SNPs: sum(correlation)/#SNPe; ρ_2_ = sum(correlation)/#SNPs

Interestingly, SHAPEITped–minimac outperformed all other population-based approaches for all MAF bins. This great performance increase was more striking for rare variants, where GIGI was also outperformed, slightly, by approximately 0.02. For this bin of MAF, BEAGLE–BEAGLE was the worst. Note that all population-based approaches performed similarly for common variants. Moreover, as expected, these approaches improved with increasing MAF, unlike GIGI whose performance slightly decreased. In these admixed pedigrees, our results also showed that MaCH–Admix (which allows for admixture), is better than MaCH–MaCH (does not allow for admixture) for rare and uncommon variants, where the difference of imputation accuracy was approximately 0.1. For common variants, both approaches led to similar results. This result stresses the need, in such data, of using imputation programs that allow for admixture in order to improve imputation accuracy.

Surprisingly, MaCH–minimac consistently outperformed MaCH–MaCH. Despite the claim that minimac and MaCH use same imputation algorithms, our results suggest otherwise, and that minimac’s undocumented algorithm may be better. To investigate this result and to determine if the difference might be a result of possible bias in calling the sequence data (eg, if sequence calling was performed using part of the minimac algorithm), we simulated sequence data on the same GAW19 pedigrees but used European ancestry data. We performed analysis with MaCH–MaCH, MaCH–minimac, SHAPEITped–minimac, and GIGI. Interestingly, GIGI performed better than all approaches for rare variants on these data (*ρ*_1_ = 0.23, 0.27, 0.39, and 0.54, respectively). In addition, MaCH–minimac performed better than MaCH–MaCH, which means that minimac’s imputation algorithm still may be better than the one used in MaCH. The underperformance of GIGI in the GAW19 data for rare variants, and possibly also the common variants, might be the result of using an admixed population. In fact, when GIGI is not able to impute genotypes using pedigree information, it draws them from the pre-specified population MAF. If these frequencies are inaccurate, resulting poor imputation is likely.

In the GAW19 Mexican American pedigrees, there are different amounts of admixture across pedigrees, suggesting the need for using pedigree-specific allele frequencies to improve GIGI’s imputation. To investigate this, we explored GIGI’s accuracy per pedigree depending on the admixture level estimated in Blue et al. [18]. However, we did not observe any correlation between admixture in pedigrees and GIGI’s accuracy (results not shown). Another possible explanation is that undetected Mendelian consistent errors, which were not in the simulated genotype data, could have led to poor IV estimation and hence poor imputation performance.

We also applied our framework to combine family- and population-based imputation data. We combined imputation results from GIGI and three 2-step population-based imputation approaches: (a) SHAPEITped–BEAGLE, (b) SHAPEITped–minimac, and (c) SHAPEITped–IMPUTE2. Overall, we show a slight improvement in imputation accuracy from using the combined data, for rare and uncommon variants, but less for common variants. This trend could be seen by the 2 correlation measures, but especially by *ρ*_2_. This measure reflects the total amount of imputation information we can glean from the 2 different sources of correlation in the data. In addition, combining both population- and pedigree-based approaches resulted in an increase in the number of imputed SNPs that are polymorphic in the sample.

### Influence of GIGI-Pick

**Fig. 1:**
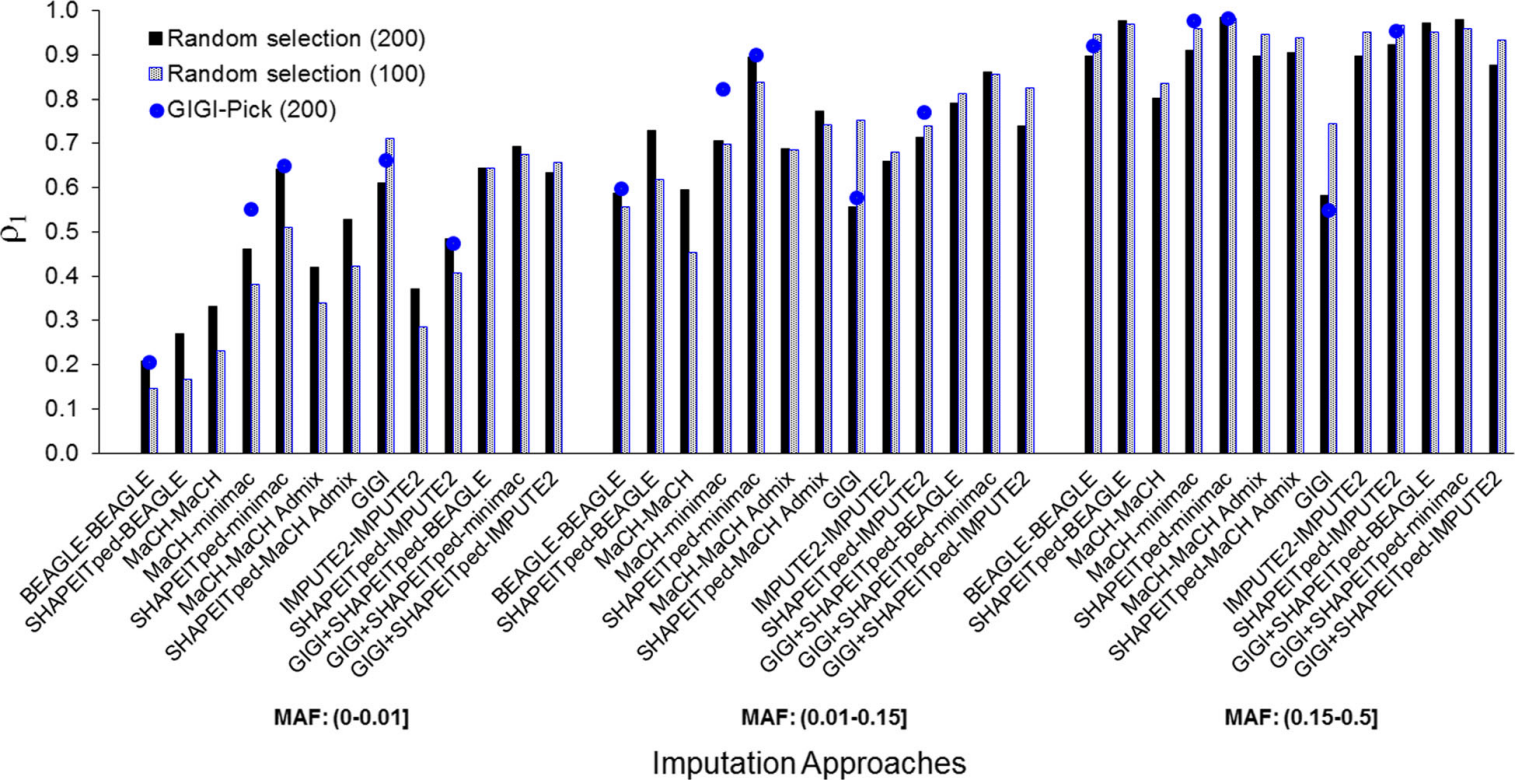
Mean correlations between imputed and reference panels from different imputation approaches. Individuals were selected randomly or via GIGI-Pick. The different imputation approaches for the 3MAF bins (ie, (0–0.01], (0.01–0.15], and (0.15–0.5] are on the *x*-axis. Mean correlations (*ρ*_*1*_) are on the *y*-axis

The selection of reference panel individuals using GIGI-Pick showed a slight increase in imputation accuracy compared to random selection, for the subset of approaches we used (Fig. 1). For all MAF bins, MaCH–minimac improved the most through use of GIGI-Pick. GIGI also improved consistently across MAF bins, especially for rare variants where it previously was shown to perform well [3]. Also, as shown in Fig. 1, we observed relatively better imputation accuracy with GIGI using smaller numbers of reference panel individuals across all MAF bins. An explanation for this better accuracy might be a decrease of Mendelian consistent errors when fewer reference panels are used. For population-based imputation, as expected, accuracy was better for higher numbers of reference panel individuals for rare and uncommon variant bins. However, accuracy was worse for common variants. This is likely explained by the decrease of phasing performance in the sparse panel individual data set when its size decreases.

### IBD mapping analysis

**Fig. 2:**
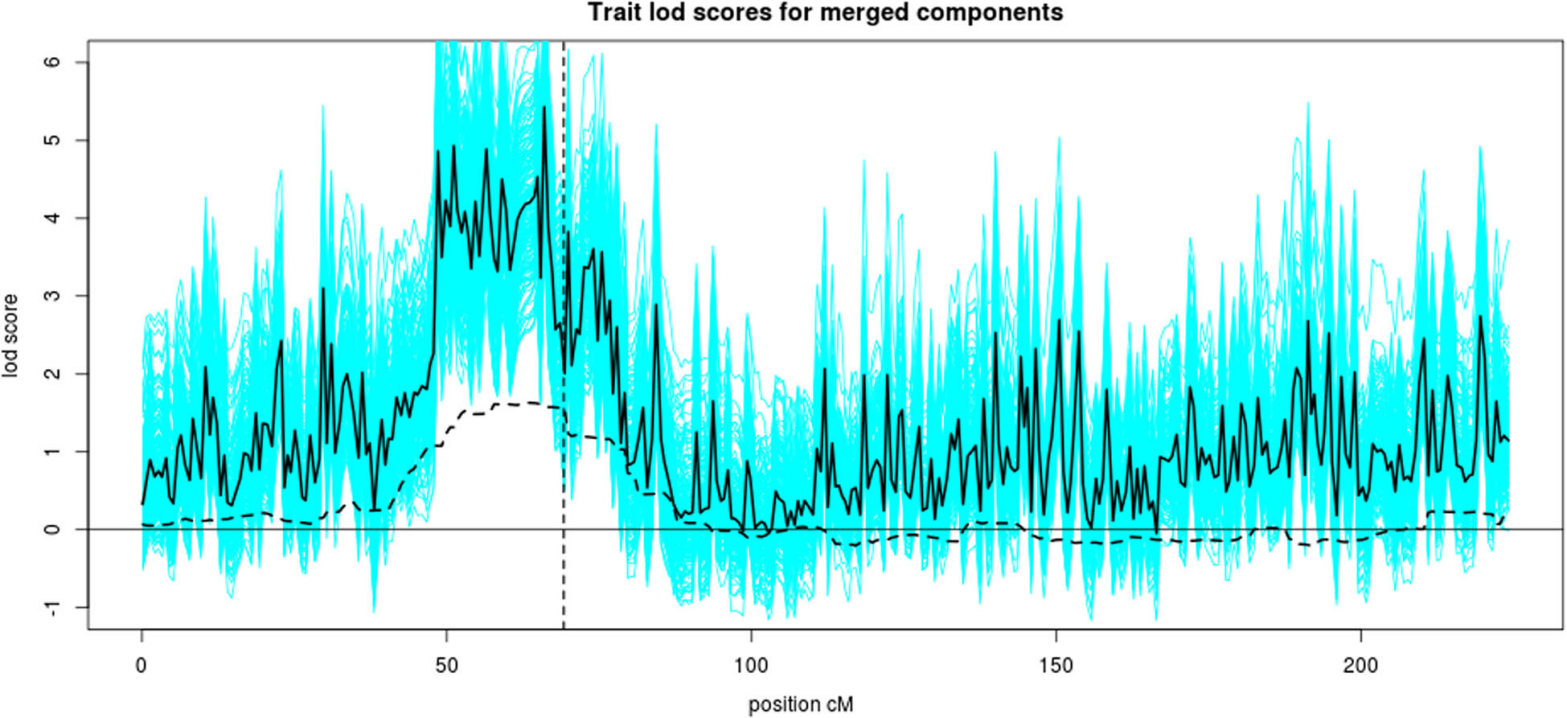
LOD score curves for merged IBD graphs for all 200 simulated traits (*cyan lines*) with their average (*solid black line*), and the average LOD score for the unmerged graphs (*dashed black line*). The location of the *MAP4* gene is indicated by the vertical line

Figure 2 shows the results of the LOD score analyses with the pedigree only and the merged population and pedigree IBD. For the unmerged IBD, there were no strong linkage signals (average LOD score is slightly higher than 1). Interestingly, adding the additional IBD inferred between the pedigrees with the merging process resulted in a much stronger linkage signal in the trait region from 50 to 75 cM where the average LOD score exceeds 3. The results show that significant extra information can be gained by merging IBD inferred jointly from denser MS-3 SNPs among more remotely related individuals with that inferred in known pedigrees using sparse MS-2SNPs.

## Discussion and conclusions

We compared the performance of several imputation approaches in pedigree data provided by GAW19 organizers. Also, we proposed and evaluated the performance of a new IBD mapping approach that combines IBD information from both unrelated and related individuals, by pedigrees, in order to identify genes implicated in complex traits. We showed that using the SHAPEIT program for pre-phasing, with its option that handles pedigree structure, along with the imputation program minimac, led to the best imputation performance for both rare and common variants. This population-based imputation approach outperformed GIGI (a family-based imputation method) not only for common variants but also for rare variants. This result was not expected for rare variants, and might be specific to this admixed data set as indicated by our results from simulated sequence data derived from European samples. Beside this specific result, most of the remaining results observed in GAW19 data were generally consistent with what we observed in our earlier simulations, which makes the results generalizable. On the other hand, our new IBD mapping approach shows promise, as it appeared to perform better than classic linkage analysis that uses only known related individuals in pedigrees.
